# Supplementary material for: MiR-23a Regulates Skin Langerhans Cell Phagocytosis and Inflammation-Induced Langerhans Cell Repopulation
Source: Biology (Basel). 2023 Jun 28;12(7):925. doi: 10.3390/biology12070925 (PMC10376168; doi:10.3390/biology12070925)
Supplement: Supplementary file 1 [file biology-12-00925-s001.zip › biology-2308981-supplementary.pdf]

Supplemental Figure S1

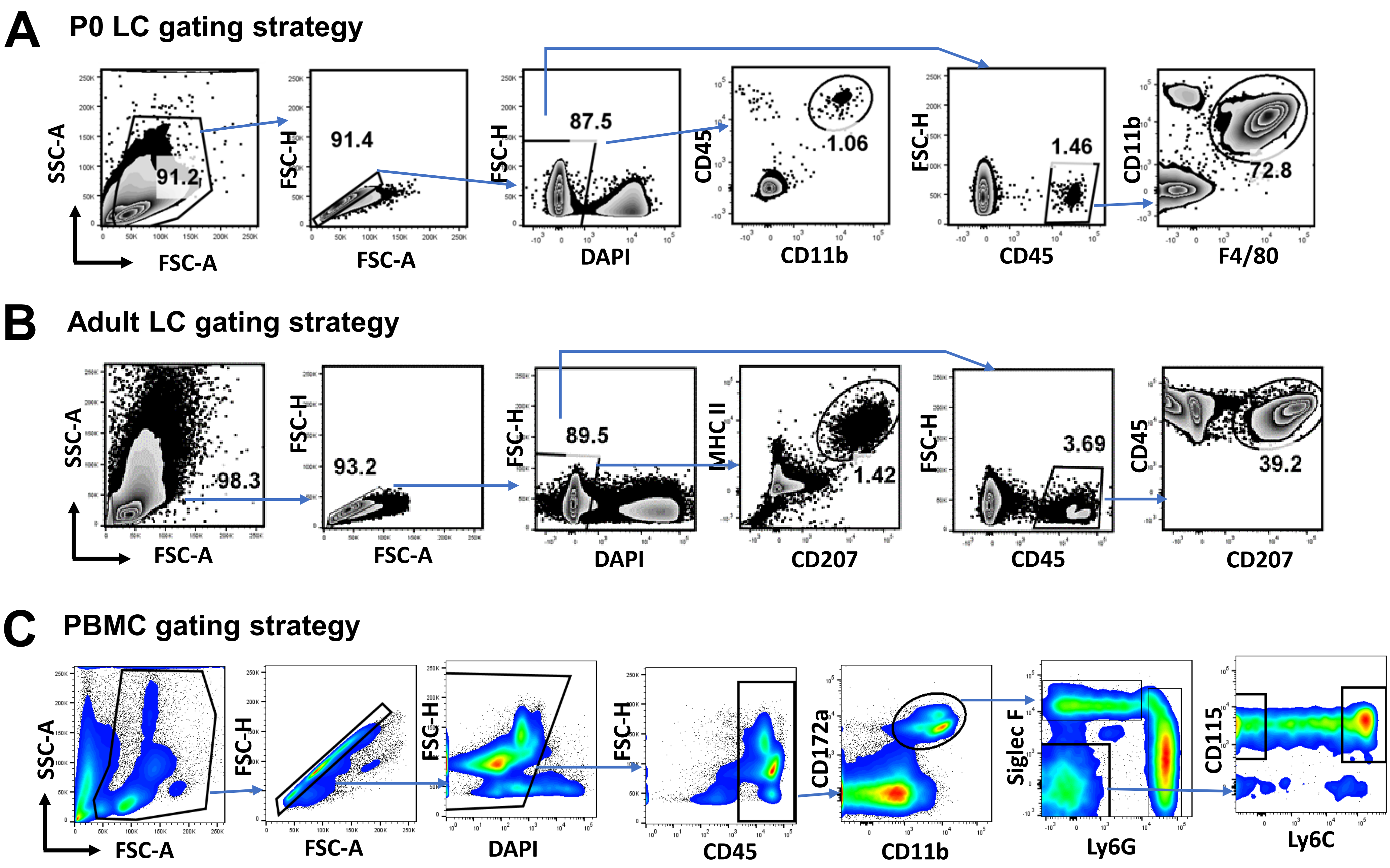

Supplemental Figure S1. Flow cytometry gating strategy for P0 epidermal LCs (A), adult epidermal LCs (B), and peripheral blood monocytes (C).

Supplemental Figure S2

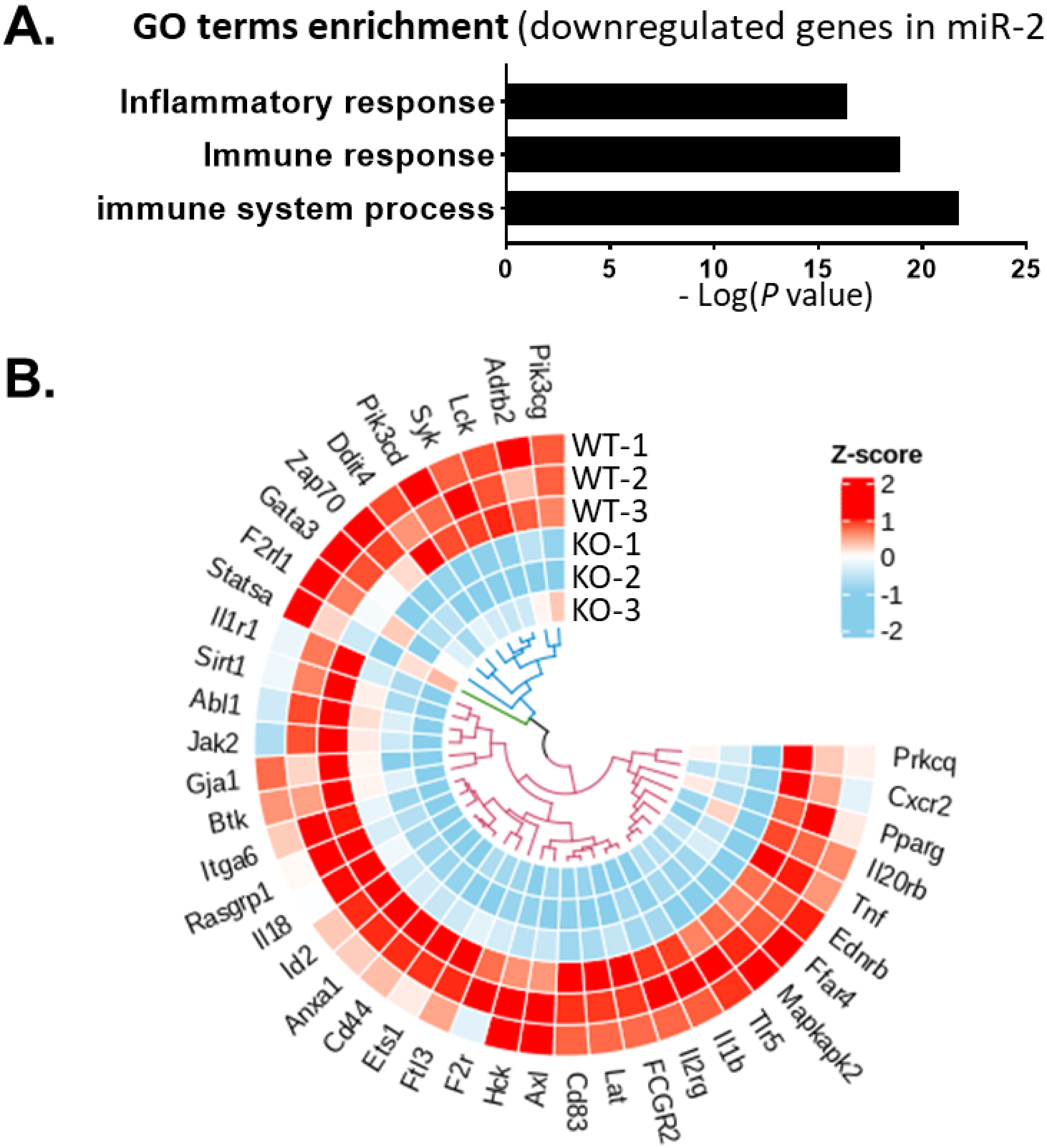

Supplemental Figure S2. Down-regulated inflammatory response related genes in steady-state LCs with miR23a deletion. (A) Gene ontology (GO) biological process enrichment analysis on the down-regulated genes in steady state miR23a KO LCs. **(B)** Heatmap showing the paired gene expression in the GO terms (A) in WT and miR23a KO LCs at steady state.

Supplemental Figure S3

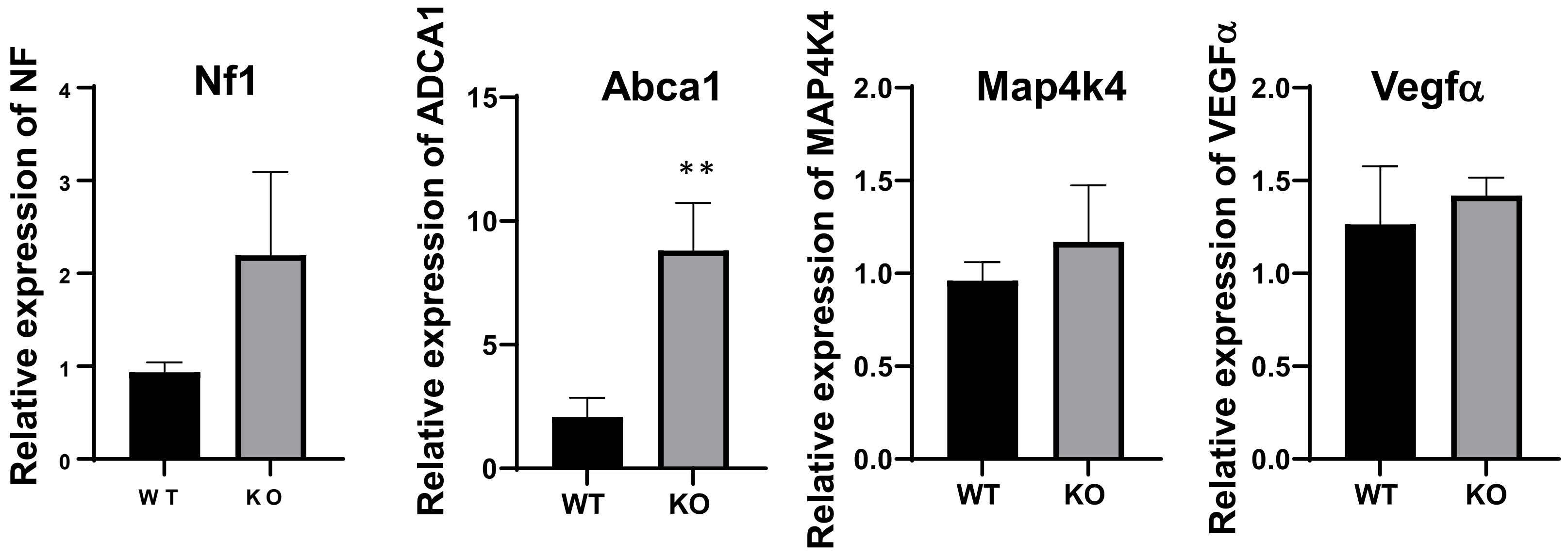

Supplemental Figure S3. PT-PCR validation of upregulated miR23a target genes in miR23a KO Langerhans cells. Four genes (Nf1, Abca1, Map4k4 and Vegfα) showed upregulation in miR23aKO LC detected by RNA-seq as Potential miR23a targets were selected to be validated using RT-PCR. Bars represent mean ± SD. **P < 0.01
